# Rsite: a computational method to identify the functional sites of noncoding RNAs

**DOI:** 10.1038/srep09179

**Published:** 2015-03-17

**Authors:** Pan Zeng, Jianwei Li, Wei Ma, Qinghua Cui

**Affiliations:** 1 Department of Biomedical Informatics, MOE Key Lab of Cardiovascular Sciences, School of Basic Medical Sciences, Peking University, 38 xueyuan Rd, Beijing. 100191, China; 2 Lab of Translational Biomedicine Informatics, School of Computer Science and Engineering, Hebei University of Technology, 5340 Xiping Rd, Tianjin. 300401, China

## Abstract

There is an increasing demand for identifying the functional sites of noncoding RNAs (ncRNAs). Here we introduce a tertiary-structure based computational approach, Rsite, which first calculates the Euclidean distances between each nucleotide and all the other nucleotides in a RNA molecule and then determines the nucleotides that are the extreme points in the distance curve as the functional sites. By analyzing two ncRNAs, tRNA (Lys) and Diels-Alder ribozyme, we demonstrated the efficiency of Rsite. As a result, Rsite recognized all of the known functional sites of the two ncRNAs, suggesting that Rsite could be a potentially useful tool for discovering the functional sites of ncRNAs. The source codes and data sets of Rsite are available at http://www.cuilab.cn/rsite.

RNA molecules are critical for a lot of cellular processes. Besides protein coding RNAs (mRNAs), recently the application of high-throughput transcriptome detection technologies has resulted in large sets of noncoding RNAs (ncRNAs)^1^,^2^, which don't have the ability of coding proteins but directly carry out functions at RNA level. More recently, increasing evidence has shown that ncRNAs have important and diverse functions^3^. Therefore, it will be not surprised that the dysfunctions of ncRNAs are associated with a wide spectrum of diseases^4^,^5^,^6^. According to the human microRNA (miRNA) disease database (HMDD, http://www.cuilab.cn/hmdd) and the lncRNA disease database (LncRNADisease, http://www.cuilab.cn/lncrnadisease), there are already ~400 diseases and ~200 diseases having been reported to be associated with miRNAs and long ncRNAs (lncRNAs), respectively. It is believed that ncRNAs are becoming a large class of novel molecules for disease diagnosis and therapy. Given the rapidly growing numbers of ncRNAs, identifying the functional sites of ncRNAs has become an important and emergent task in ncRNA research field. However, there is a paucity of methods.

For the above purpose, recently, some biological-experiments based pioneering methods have been developed, such as SHAPE-MaP (SHAPE and mutational profiling)^7^, RNase footprinting^8^,^9^, and in-line Probing^10^,^11^. Although the above methods provide great helps in RNA research, they have some inherent limitations. First, these biological experiments are generally complex and thus time and cost consuming. Moreover, the above methods work on a level of RNA backbone or RNA domain, so they have difficulties in identifying base-level functional sites. Therefore, computational methods will be an important supplement to the above biological-experiments based methods. However, no computational methods for the above purpose have been developed so far.

For proteins, one class of much more well-studied biological molecules than ncRNAs, there are already a number of computational methods to predict their functional sites, including methods based on machine learning^12^, sequence conservation^13^, and tertiary structure^14^. These methods provide clues for the prediction of ncRNA functional sites. For machine learning based methods and sequence conservation based methods, prior known protein functional sites are needed. Because ncRNAs represent a new research field, the known ncRNA functional sites are limited. Moreover, ncRNAs normally show less sequence conservation. In addition, the conserved bases are not often the functional sites and the divergent bases are not often the non-functional sites. For example, divergent bases of one RNA across various species could be the functional sites indicating the functional divergence of the RNA in different species. Therefore, currently these methods are not feasible for the identification of ncRNA functional sites. For the tertiary structure based methods, there are two main categories: those predicting functional sites by structural similarities from proteins with known functional sites and those predicting functional sites by structural features such as geometry or electrostatics^15^. The first category also requires datasets of known protein functional sites. This category is thus not practicable for ncRNAs because a resource for ncRNA functional sites is not available yet. For the geometry based method, the residues that show high closeness centrality normally are considered to be those involved in function^14^. For example, it was reported that catalytic residues tend to be close to the molecular centroid^16^. Notably, geometry based methods are not dependent on known functional sites. Given limited known functional sites of ncRNAs, geometry based methods seem feasible to predict ncRNA functional sites. Moreover, in addition to the central residues described above, the surface-exposed ones could also be the functional sites of molecules^17^. Taken together, we hypothesized that both the most connected nucleotides and the most non-connected nucleotides in an ncRNA molecule tend to be functionally important, that is, they are putative functional sites.

Based on the above observations, here we presented a computational method, Rsite, for the identification of ncRNA functional sites based on ncRNA geometry. The results showed that Rsite has a reliable accuracy, suggesting that Rsite could be a useful tool for the identification of ncRNA functional sites.

## Results

In order to validate the accuracy and efficiency of Rsite, we applied it to two well-studied ncRNAs with known functional sites, tRNA (Lys) and Diels-Alder ribozyme.

### The functional sites of the tRNA (Lys)

**Figure 1:**
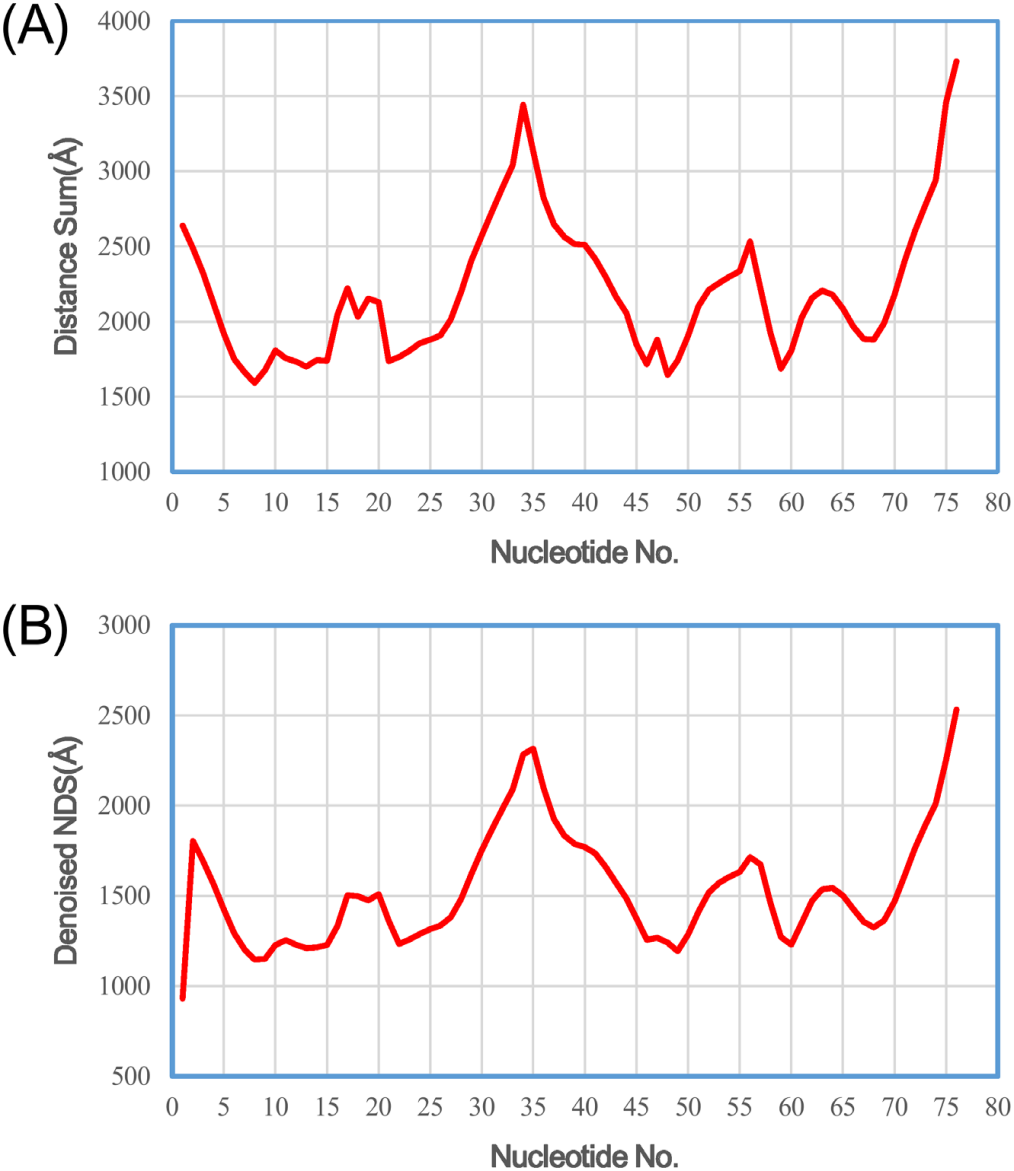
The raw nucleotide distance curve (A) and the smoothened nucleotide distance curve (B) of the tRNA (Lys).

**Figure 2:**
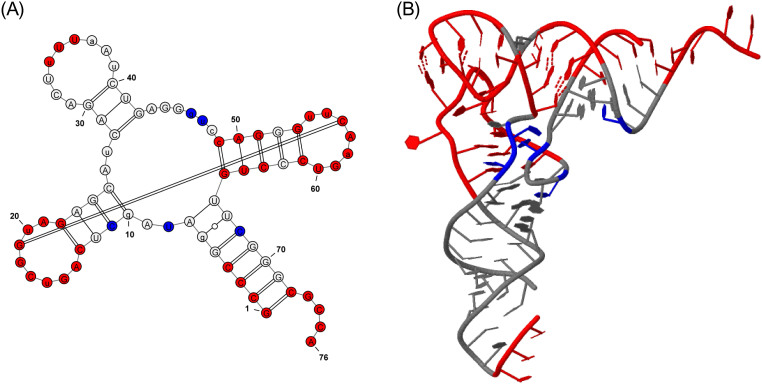
Graphical representation of the tRNA (Lys) functional sites identified by Rsite. Figure 2A&B show the secondary structure and tertiary structure of the tRNA (Lys). The nucleotides in red color represent the predicted functional sites that hit known functional sites. The nucleotides in blue color stand for the predicted functional sites that are not reported to be functional sites.

We first applied Rsite to predict the functional sites of the tRNA (Lys). Rsite first calculated the distance curve of the tRNA (Lys) (Figure 1). Rsite next identified the putative functional sites of the tRNA (Lys). As a result, all the 7 known functional sites were successfully predicted by Rsite (Table 1; Figure 2), which achieved a sensitivity of 100% (7/7). Among all the 11 predicted functional sites, 4 sites were not reported to be functional sites, suggesting that Rsite has a low false positive rate.

### The functional sites of the Diels-Alder ribozyme

**Figure 3:**
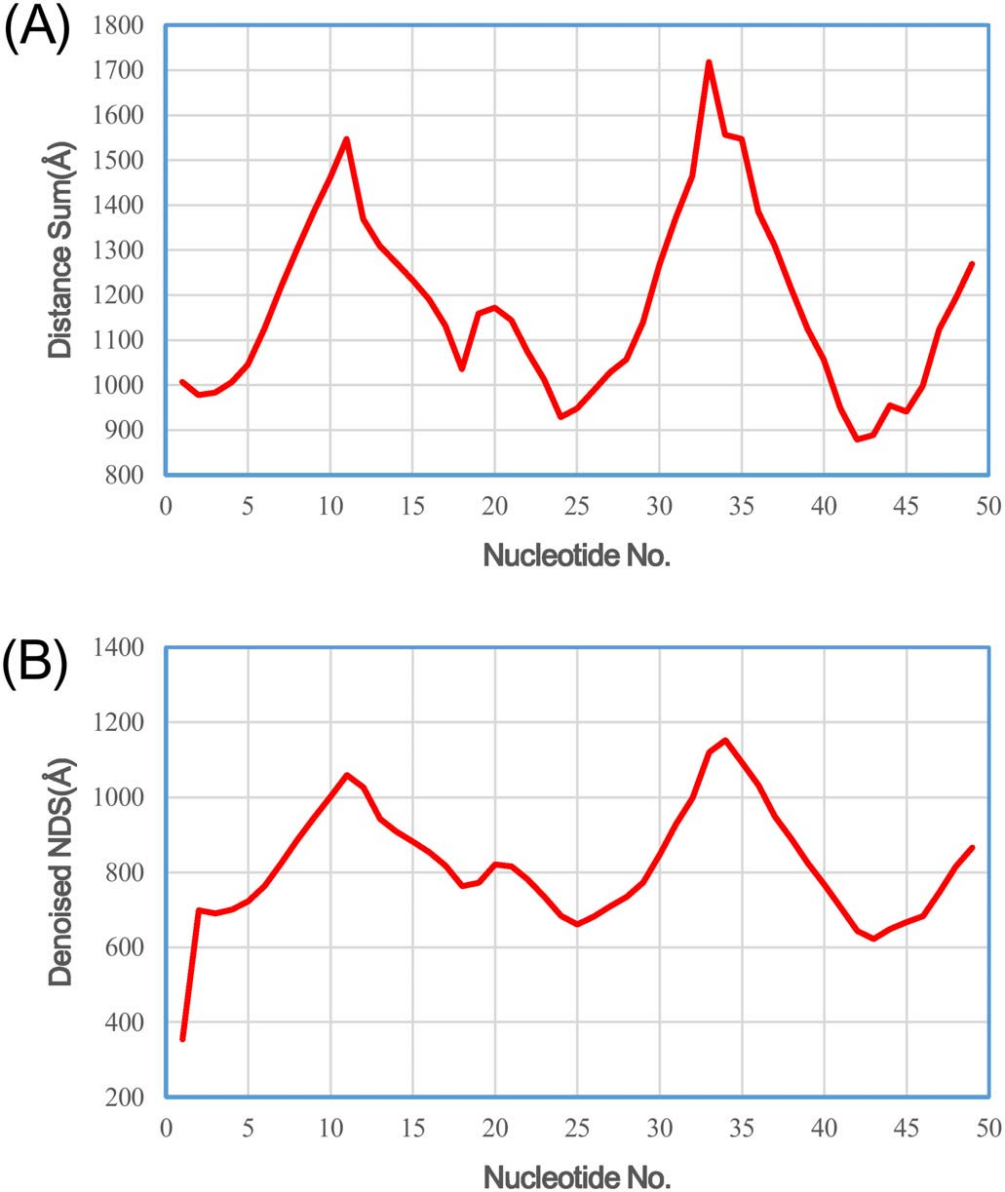
The raw nucleotide distance curve (A) and the smoothened nucleotide distance curve (B) of the Diels-Alder ribozyme.

**Figure 4:**
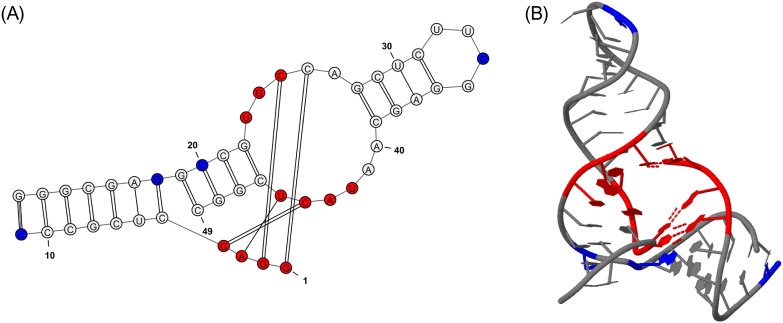
Graphical representation of the Diels-Alder ribozyme functional sites identified by Rsite. Supplementary Figure 2A&B show the secondary structure and tertiary structure of the Diels-Alder ribozyme. The nucleotides in red color represent the predicted functional sites that hit known functional sites. The nucleotides in blue color stand for the predicted functional sites that are not reported to be functional sites.

We also applied Rsite to predict the functional sites of the Diels-Alder ribozyme. Rsite first calculated the distance curve of the Diels-Alder ribozyme (Figure 3). Rsite next identified the putative functional sites of the Diels-Alder ribozyme. As a result, Rsite successfully predicted all the 3 known functional sites (Table 2; Figure 4), indicating Rsite also achieved a sensitivity of 100% for the Diels-Alder ribozyme. Among the 6 predicted functional sites, 3 sites were not reported to be functional sites.

## Discussion

As demonstrated by the above analyses, Rsite shows a reliable accuracy for the identification of ncRNA functional sites if the ncRNAs' tertiary structures are available, which would shed light on the ncRNA research. Currently, Rsite is only designed to predict the positions of functional sites but cannot predict their exact functional roles. Although this limitation exists, we think it is still helpful and useful because it presents molecular biologists candidate targets for further experiments. Combining molecular biology, we believe it could be feasible to investigate the exact functional roles of the identified functional sites.

Given that the tertiary structures of some ncRNAs can be predicted by computational tools, Rsite can be easily applied to a number of ncRNAs based on predicted tertiary structures. However, the current algorithms predicting RNA tertiary structures runs well only for small RNAs (e.g. RNAs less than 100 nt in length) but cannot process bigger RNAs. This limits Rsite to a small fraction of ncRNAs. Therefore, doing as the said above must be very careful because of poor prediction accuracy of tertiary structures for RNA molecules, especially for large RNA molecules. Moreover, we believe that combining with other features such as sequence conservation can further improve Rsite. In addition, validation with more structures will help establish utility of Rsite. The NUCLEIC ACID DATABASE (NDB, http://ndbserver.rutgers.edu) collects a number of RNA tertiary structures^18^. However, currently, these RNAs do not have detailed information about the annotation of functional sites. It is thus necessary to re-visit the NDB database to test the validation of Rsite with more RNAs when their functional sites become available. Another limitation of Rsite is that it is difficult to predict the exact functional domains of a RNA molecule. The reason is that Rsite tries to find the local extreme points but misses the neighbor points of the extreme points. This procedure often successfully identifies one functional site located within a functional domain but misses the other functional sites in this domain. In the future, integrating the neighbor points of an extreme point could be an optional solution for this problem. Finally, although limitations exist, we envision that Rsite represent a potentially useful tool for biologists working on ncRNA research.

## Methods

### The tertiary structure data of two ncRNA molecules

To validate the accuracy of Rsite, we applied it to two ncRNA molecules, tRNA (Lys) and Diels-Alder ribozyme. We obtained the tertiary structure data of the two ncRNAs from the PDB database (http://www.rcsb.org/pdb/home/home.do) (PDB ID of the tRNA (Lys): 1FIR; PDB ID of the Diels-Alder ribozyme: 1YKV). The tertiary structure data of the two ncRNAs are also available at http://www.cuilab.cn/rsite.

### Algorithms in Rsite

For a given ncRNA, Rsite first calculates the Euclidean distance between any two nucleotides using the coordinates of the two nucleotides derived from the ncRNA's structure data. Then for each nucleotide, Rsite sums up the distances between the nucleotide and all the other nucleotides. For an ncRNA with *n* nucleotides, we then obtain a distance curve (*D*) of length *n*. Here *D(i)* denotes the summed distance between the *ith* nucleotide and all the other nucleotides. Next, in order to decrease noise, the distance curve is smoothened by a Gaussian filter. For this purpose, here we used a window size of 2 for both RNAs. It is difficult to determine an optimal window size, which could have important influence on the prediction result. For longer RNAs, a bigger window size could be better. The local maximum points and the local minimum points of the smoothened distance curve are then identified. Finally, the extreme points are identified as the functional sites of the ncRNA. For the start point and the end point, if they show relative high (50 percentage) deviation from the average distance they will be considered to be functional sites. In addition, because most of the functional sites identified by Rsite are single nucleotide, we further merge multiple functional sites into one if they are close (< = 2 nucleotides for their sequence positions) to each other.

## Author Contributions

Q.C. designed this study. P.Z. performed the study. J.L. and W.M. contributed to the coding of Rsite. Q.C. and P.Z. wrote the manuscript.

## Figures and Tables

**Table 1:** The known functional sites (FSs) and predicted results on the tRNA(Lys,3)(PDB#1FIR)

Site No	FS	Predicted FS	Description
**1**	1–4	1,2	Within acceptor stem(5' end)Interacting with RanContacting XpotInvolved in recognition by RNase Z and RNase PRecognized by aminoacyl-tRNA synthetase
**2**	13–22	13,17,19,20,22	DHU loopInteracting with the mRNA-ribosome complexContacting XpotInvolved in recognition by RNase P
**3**	34–36	35	Anticodon, Decoding mRNA codonRecognized by aminoacyl-tRNA synthetaseInteracting with the mRNA-ribosome complex
**4**	49–51	49	Within TψC stemBinding site of elongation factor
**5**	53–61	56,60	TψC loopInteracting with the mRNA-ribosome complexContacting XpotInvolved in recognition by RNase Z and RNase PProcessed by a tRNA ψ55 pseudouridine synthaseAffect 3' end processing and tRNA structure
**6**	63–65	64	Within TψC stemBinding site of elongation factorInteracting with RanInvolved in recognition by RNase P
**7**	72–76	76	Aminoacylation site(3' end)Recognized by aminoacyl-tRNA synthetaseInteracting with the mRNA-ribosome complex (7)Contacting Xpot (1)Involved in recognition by RNase ZProcessed by a CCA-adding enzyme

**Table 2:** The known functional sites (FSs) and predicted results on the artificial Diels-Alder ribozyme(PDB#1YKV)

Site No	FS	Predicted FS	Description
**1**	1–4	1,2,3	A part of the catalytic pocket
**2**	23–25	25	A part of the catalytic pocket
**3**	42–45	43	A part of the catalytic pocket
